# Editorial: Metabolic consequences in children and adolescents with obesity: latest insights

**DOI:** 10.3389/fendo.2023.1223129

**Published:** 2023-05-31

**Authors:** Joanna Helena Sliwowska, Malgorzata Wojcik, Marwan El Ghoch, Maurizio Delvecchio

**Affiliations:** ^1^ Laboratory of Neurobiology, Institute of Zoology, Poznań University of Life Sciences, Poznań, Poland; ^2^ Department of Pediatric and Adolescent Endocrinology, Chair of Pediatrics, Pediatric Institute, Jagiellonian University Medical College, Kraków, Poland; ^3^ Department of Nutrition and Dietetics, Faculty of Health Sciences, Beirut Arab University, Beirut, Lebanon; ^4^ Faculty of Medicine, UniCamillus – Saint Camillus International University of Health and Medical Sciences, Rome, Italy; ^5^ Metabolic Disorder and Diabetes Unit, "Giovanni XXIII" Children Hospital, Bari, Italy

**Keywords:** metabolic syndrome, type 2 diabetes mellitus, obesity, metabolic complications, BMI - body mass index, globesity

Over the last decades, our environment, behaviors and lifestyle have significantly changed. The consumption of unhealthy food, rich of calories and highly palatable, prompts children and adolescents to overeating. Moreover, reduction in physical activity levels has occurred with a significant effect on health and wellbeing.

The escalation of obesity prompted the World Health Organization (WHO) to adopt in 2001 a new term, “globesity” which includes “global” and “obesity” and indicates the spread of obesity in all the continents and underlines how much this problem has become relevant. As well as the prevalence of obesity in children and adolescents continues to increase, weight-related complications, mostly cardiovascular ones, are expected to increase in these age groups (1, 2). In this Research Topic we aimed to collect high-quality papers focusing on metabolic consequences. Six papers, 2 reviews, and 4 original research papers, were published.

The mini review by Oboza et al. aimed to find answers to the question: Can type 1 diabetes (T1D) be an unexpected complication of obesity? T1D is a chronic autoimmune disease in which insulin deficiency is observed. The authors discuss pathogenesis, environmental risk factors (overweight and obesity), and the development of obesity in T1D patients. They present cases of T1D with obesity and insulin resistance, classified as ‘double diabetes’. It is concluded that there is convincing evidence indicating that T1D may be a complication of obesity. Furthermore, due to a significant increase in obesity among children and adolescents, it is predicted that shortly, there will also be a rise in the number of cases of T1D. Thus, a global attempt to address this medical condition is needed in order to prevent obesity in children as well as in adolescents.

In the second mini review Tagi et al. focused their attention on insulin resistance, which plays a key-role in the development of obesity-related consequences. A 2010 International Consensus Statement (3) aimed to define insulin resistance in childhood, its risk factors, and how it can be measured, prevented and treated, in order to avoid the possible long-term consequences. Some points are still discussed, such as insulin sensitivity measurement methods and its standardization. Furthermore, the evidence regarding possibilities of the treatment of insulin resistance with lifestyle changes and medications, recently available for clinical use only in the last years, need to be updated. This paper provides a useful overview of the most recent evidence on these points and it may support the clinicians in their clinical practice and the researchers in the development of future studies.

In adults, obesity is associated with several metabolic alterations, which have been identified as features of a disease named metabolic syndrome (MetS). In their study, Liu et al. aimed to investigate the performance of two obesity indicators, the visceral adiposity index (VAI) and the lipid accumulation product (LAP) on MetS in young adults. They recruited 448 subjects, 19-24 years old, and evaluated the accuracy of these two indicators for MetS. In this sample study, 2% of the subjects were diagnosed with MetS and the performance of these indicators was similar. They conclude that both of them are useful to screen for MetS.

Weight change is a risk factor for MetS. Dai et al. aimed to evaluate the risk for MetS in adults with normal weight and overweight or obesity. They enrolled 1895 subjects and evaluated their weight in 2012 and 2020. Participants were stratified into five different subgroups, on the basis of weight change. They found out that the relative risk of developing metabolic abnormalities increased by 22% every kilogram increase over the study period. A weight gain above 4 kg has a detrimental effect only in subjects in normal weight, but not those with overweight or obesity. Interestingly, a weight loss of at least 4 kg reduces the risk of metabolic abnormalities only in subjects with overweight or obesity. A similar study conducted by Wang et al. in 437,849 participants of 35-64 years old with body weight recall at 25 years. The authors showed an increase of MetS of 2.01, 1.93 and 1.67 every 5 kg increase from underweight to normal weight, to overweight or obesity. As result, both of these studies concluded that weight gain increases the risk of metabolic abnormalities in normal weight participants. On the other hand, weight maintenance and weight loss mitigate and reduce the risk for metabolic abnormalities in individuals with overweight or obesity.

Finally, Chen et al. explored the role of the relationship between effect of maternal lipid levels on birthweight and the long-term health of the offsprings. They recruited 705 women and their 1,410 offsprings, two for each woman. All the pregnant were between 19 and 44 years of age and all the neonates were full termed. They found that gestational age, neonatal gender, parity, maternal pre-pregnancy BMI and maternal weight gain were positively correlated with infants birthweight. However, also the total cholesterol levels of the third trimester were positively related to birthweight, with about 50 gr increase in birthweight every unit increase in total cholesterol. On the other hand, HDL cholesterol was negatively associated with birthweight. The regression analysis confirmed the predictive role of cholesterol level during pregnancy and thus the authors conclude that it is an independent factor, which may affect the birthweight of the offsprings.

In conclusion, obesity has become a worldwide health problem and its consequences are a growing field of research. It is well acknowledged that an obese child will be an adult with a larger risk of obesity and cardiovascular disease (4) and this is why preventing and treating obesity have become priority aims in several Countries. We hope that you will enjoy this Research Topic and that its papers will support you in clinical practice and in your research activities.

## Author contributions

All authors listed, have made substantial, direct, and intellectual contribution to the work, and approved it for publication.
